# Automated detection of interictal epileptiform discharges with few electroencephalographic channels

**DOI:** 10.1111/epi.18431

**Published:** 2025-05-03

**Authors:** Moritz Alkofer, Chaoqi Yang, Wolfgang Ganglberger, Jules Beal, Manu Hegde, Joon‐Yi Kang, Ji Yeoun Yoo, Michael A. Gelfand, Liu Lin Thio, Ekrem Kutluay, Zeke Campbell, Sarah Schmitt, Ezequiel Gleichgerrcht, Elizabeth Waterhouse, Maria R. Lopez, Stephan Eisenschenk, Mattia Galanti, Rani K. Singh, Kristin E. Wills, Erik‐Jan Meulenbrugge, Dennis Dlugos, Brian Dean, Jonathan J. Halford, Daniel Goldenholz, Jin Jing, Robert Thomas, M. Brandon Westover

**Affiliations:** ^1^ Neurology Department, Harvard Medical School Beth Israel Deaconess Medical Center Boston Massachusetts USA; ^2^ Institute for Theoretical Physics Technische Universität Berlin Berlin Germany; ^3^ Computer Science Department University of Illinois at Urbana Champaign Urbana Illinois USA; ^4^ Department of Pediatrics Weill Cornell Medical College New York New York USA; ^5^ Weill Institute for Neurosciences University of California, San Francisco San Francisco California USA; ^6^ Department of Neurology Johns Hopkins School of Medicine Baltimore Maryland USA; ^7^ Department of Neurology Icahn School of Medicine at Mount Sinai New York New York USA; ^8^ Department of Neurology Penn Medicine Philadelphia Pennsylvania USA; ^9^ Department of Neurology Washington University St. Louis Missouri USA; ^10^ Neurology Department Medical University of South Carolina Charleston South Carolina USA; ^11^ Department of Neurology Ralph H. Johnson Department of Veterans Affairs Medical Center Charleston South Carolina USA; ^12^ Department of Neurology Emory University Hospital Atlanta Georgia USA; ^13^ Richmond VA Medical Center Richmond Virginia USA; ^14^ Bruce W. Carter Department of Veterans Affairs Medical Center Miami Florida USA; ^15^ University of Miami Miami Florida USA; ^16^ Department of Neurology University of Florida Gainesville Florida USA; ^17^ Malcom Randall Department of Veterans Affairs Medical Center Gainesville Florida USA; ^18^ Department of Neurosciences Clemson University Clemson South Carolina USA; ^19^ Department of Public Health Sciences, College of Medicine Medical University of South Carolina Charleston South Carolina USA; ^20^ Department of Clinical Neurophysiology Children's Hospital of Philadelphia Philadelphia Pennsylvania USA; ^21^ Department of Medicine, Harvard Medical School Beth Israel Deaconess Medical Center Boston Massachusetts USA

**Keywords:** EEG, epilepsy, interictal epileptiform discharges, machine learning, neurology, wearables

## Abstract

Interictal epileptiform discharges (IEDs) are crucial for epilepsy diagnosis and management. New electroencephalographic (EEG) devices with fewer electrodes are more accessible, but their ability to detect IEDs is uncertain. The aim of this study is to determine whether IEDs can be reliably detected in reduced‐channel EEG data, enabling broader epilepsy diagnosis. Using EEG samples from 3378 patients and an external validation set of 51 patients, we trained Cyclops, a deep neural network designed to function across various channel configurations. Performance was evaluated using area under the receiver operating characteristic curve (AUROC) and other clinically relevant metrics, including IED source location sensitivity. Cyclops demonstrated strong performance even with minimal channels. AUROC for one channel was .876 (95% confidence interval [CI] = .854–.897); best configuration based on a clinically available product was .950 (95% CI = .936–.962); for the detection of focal IEDs with two local channels, AUROC values ranged from .701 (95% CI = .656–.745) to .930 (95% CI = .902–.955), with a median AUROC of .809. On the external validation set, performance ranged from .692 (95% CI = .593–.782) to .949 (95% CI = .922–.972), with a median AUROC of .846. Thus, Cyclops demonstrates that effective IED detection is possible with reduced EEG setups, enhancing accessibility and expanding epilepsy diagnosis to broader patient populations.

## 1 INTRODUCTION

Epilepsy is a prevalent, chronic neurological disorder characterized by recurrent, unprovoked seizures. With proper diagnosis and management, up to 70% of the approximately 50 million individuals affected worldwide have the potential to live seizure‐free. However, due to the scarcity of expertise in both EEG setup and interpretation, accessing accurate diagnostic testing remains a key challenge.^1^

Epilepsy diagnosis is supported by detecting focal or generalized interictal epileptiform discharges (IEDs). Also referred to as spikes, these transient abnormal waveforms occur almost exclusively in people with epilepsy.^2^

Current practices predominantly rely on visual interpretation of clinical whole‐scalp electroencephalograms (EEGs). Prior research on reduced montages has primarily explored seizure monitoring,^3^ without addressing the potential of these systems for detecting IEDs. Studies on IED detection typically use the standard 10–20 montage.^4^ Acquiring scalp EEG with the 21 electrodes of the 10–20 standard^5^ has many advantages but is resource‐intensive and limits the use of EEG diagnostics in the third world and rural United States. Automated IED detection leveraging emerging reduced‐channel EEG devices presents an opportunity to simplify this process.

Here, we aim to bridge this gap by developing a machine learning model capable of accurately identifying IEDs with a limited number of EEG channels. We hypothesize that even highly focal IEDs may produce detectable signatures that can be captured by neighboring electrodes, making it feasible to diagnose epilepsy with simplified equipment. By potentially enabling nonspecialists to conduct preliminary assessments and facilitating wider access to diagnostic tools, this research could advance epilepsy care, especially in underserved areas.

## 2 MATERIALS AND METHODS

### 2.1 Data

We used a total of 115 454 samples from 3631 patients, (48% female, median age = 50 years; Table S1), classified by 3‐23 (median 8) independent neurologists who specialize in epilepsy and clinical neurophysiology.

The internal dataset consisted of 112 154 samples from 3580 patients and was recorded at Massachusetts General Hospital, Boston (MGH). A total of 1268 positive samples were categorized based on their source localization by one expert (M.B.W.). This dataset was divided using two strategies. A primary patientwise split of 80/10/10 for training, validation, and testing (internal validation). The test set was filtered for strong interrater agreement. IEDs with “strong interrater agreement” are samples where most experts (≥7/8) agree on the presence of an IED. To investigate the influence of rater agreement thresholds, multiple thresholds were evaluated in Figure S8. A secondary split addressed the scarcity of localized samples, directing them exclusively to the test set. Data annotation and splitting are described in the supplemental material. Note that internal validation observed strict separation at the patient level between training and testing sets, with no overlapping patients between model development (training) and testing data.

The external dataset was derived from EEG recordings exceeding 1 h each, taken from 188 recordings within the Human Epilepsy Project (HEP), a 6‐year, prospective, observational, 29‐site multicenter study of 18–60‐year‐old patients with newly diagnosed focal epilepsy. Recordings varied from routine EEGs to approximately 24‐h EEG recordings.

### 2.2 Standard protocol approvals, registrations, and patient consents

Data derived from MGH was approved by the hospital's institutional review board (IRB) with a waiver of informed consent. For the data derived from the HEP study, following local site IRB approval, patients were enrolled after obtaining written informed consent. Enrollment took place from July 18, 2012 to September 28, 2017.

### 2.3 Training

The previously described convolutional neural network^6^ was trained to predict IED probability from any potential referential EEG signal and was used to explore a wide set of montages. As depicted in Figure S3, the model was trained to predict the percentage of experts voting that a given sample contains an IED. During training, random channel values were set to 0 (not deleted, keeping the input shape constant), leading to IED representations robust to channel deletion. Data of shape 19, 128 (channels, 1 s at 128 Hz) were provided to the model in average montage, calculated using only the nonmasked channels. An individual model was trained for both dataset splits.

Each model was trained using the adaptive moment estimation optimizer (Adam), configured with a learning rate of .0001, a batch size of 128, and a weight decay of .0001. These standard parameters resulted in good performance, which did not improve significantly with tweaking. To focus on the main task, no further hyperparameter tuning was conducted. Binary cross‐entropy served as the loss function. To prevent overfitting, early stopping was employed; training ceased when the validation loss failed to decrease over a span of 20 epochs, and the model with the lowest validation loss observed up to that point was selected for use.

For data augmentation, several changes were introduced. EEG segments were randomly (1) jittered by up to ±.1 s, (2) scaled by up to ±10% amplitude, and (3) channelwise mirrored left–right. These augmentations were applied to the training dataset only. After augmentation changes, each training segment's channel amplitude was scaled by their 95th percentile and centered around its mean.

### 2.4 Evaluation methodology

First, a baseline performance level was established on the primary split by evaluating the model on samples with *n* randomly selected channels, with *n* ranging from 0 to 19.

To gauge real‐world performance, we explored reduced montages similar to those used by two commercial subscalp systems, *M1*: T3, F7, T4, and F8^7^; *M2*: T5, P3, Pz, T6, and P4^8^ as well as reduced montages used by commercially available EEG devices; *M3*: Fp1, Fp2, F7, F8, O1, and O2^9^; and *M4*: Fp1, Fp2, F7, F8, T3, T5, T4, T6, O1, and O2,^10^ as well as the channels available in typical clinical polysomnography data, *M‐PSG*: F3, C3, O1, F4, C4, and O4, and all 10–20 channels: Fp1, F3, C3, P3, F7, T3, T5, O1, Fz, Cz, Pz, Fp2, F4, C4, P4, F8, T4, T6, and O2.

The impact of IED relative to electrode location was evaluated on the secondary split. Samples from the test set were classified using regional electrode pairs, including those not proximal to the IED origin. Channels were assessed in multiple brain regions: frontal (Fp1, Fp2), central (C3, C4), parietal (P3, P4), occipital (O1, O2), and temporal (T3, T4).

### 2.5 Statistical analysis

The area under the receiver operating characteristic curve (AUROC) and 95% confidence interval (CI) were calculated using 10 000 rounds of bootstrapping. In experiments involving random channel selection, predictions for each bootstrap sample were generated using newly randomized channel sets.

## 3 RESULTS

We first explored Cyclops' capability to detect IEDs using a fixed number of random channels. Using the full montage, Cyclops achieved an AUROC of .951 (95% CI = .939–.963). When restricted to a single random channel, the model achieved an AUROC of .876 (95% CI = .854–.897), preserving 92.0% of the performance. With a configuration of four random channels, the model reached an AUROC of .920 (95% CI = .903–.937), maintaining 96.7% of the full montage performance.

**FIGURE 1:**
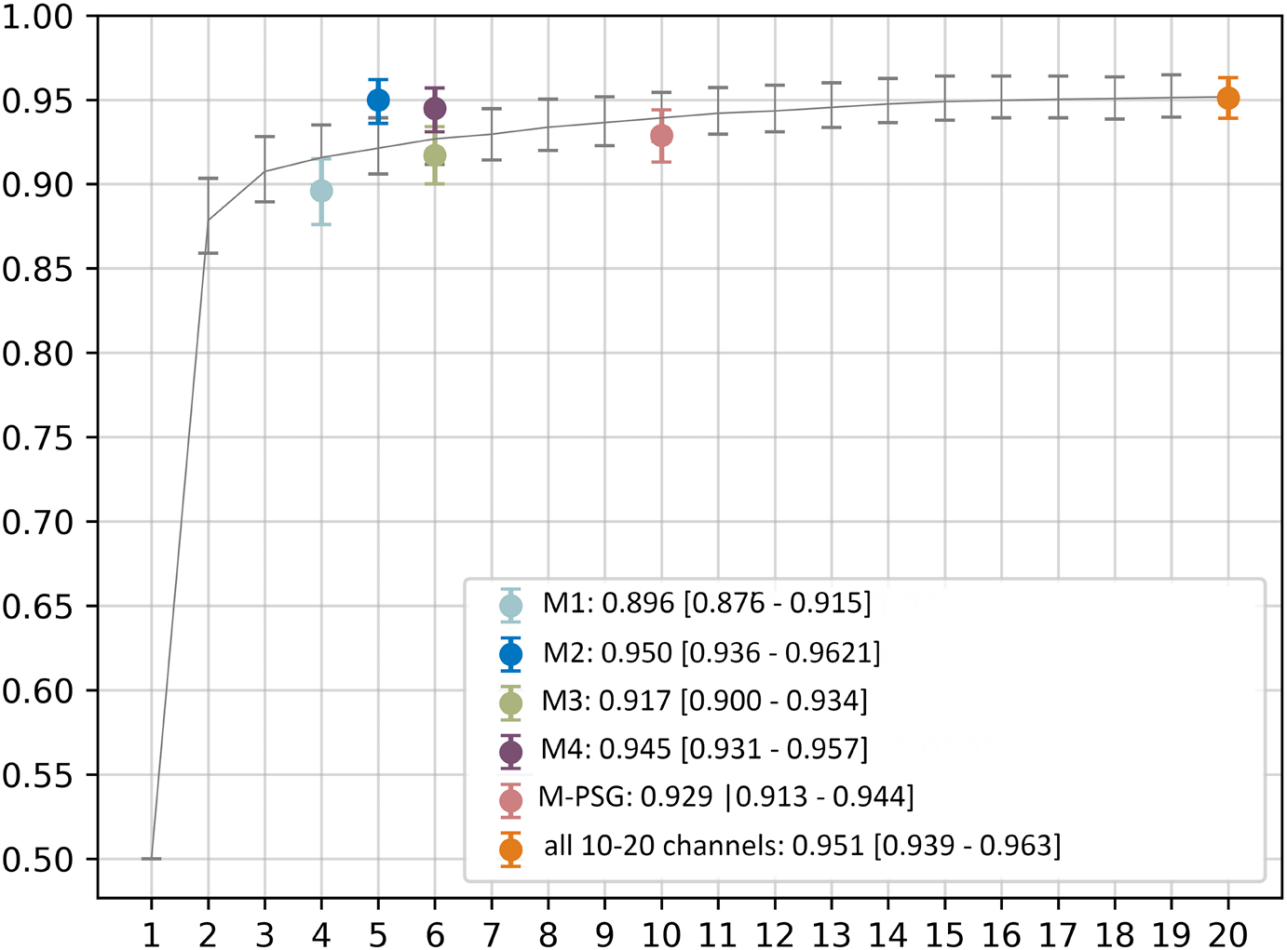
Cyclops evaluation for a range of *n* random channels as well as specific setups. The setups evaluated are *M1*: T3, F7, T4, and F8; *M2*: T5, P3, Pz, T6, and P4; *M3*: Fp1, Fp2, F7, F8, T3, T5, T4, T6, O1, and O2: *M4*: Fp1, Fp2, F7, F8, O1, and O2; M‐PSG (a standard polysomnography montage) : F3, C3, O1, F4, C4, O4: all 10–20 channels: Fp1, F3, C3, P3, F7, T3, T5, O1, Fz, Cz, Pz, Fp2, F4, C4, P4, F8, T4, T6, and O2. The area under the receiving operator curve with 95% confidence interval is used to assess the model's performance. Even with only one random channel, the model performs well. The area under the curve for random channels is .866.

The best AUROCs for configurations based on clinically available setups ranged from .896 (95% CI = .876–.915) for *M1* to .950 (95% CI = .936–.962) for *M2*, maintaining 99.9% compared to the full montage (Figure 1).

**FIGURE 2:**
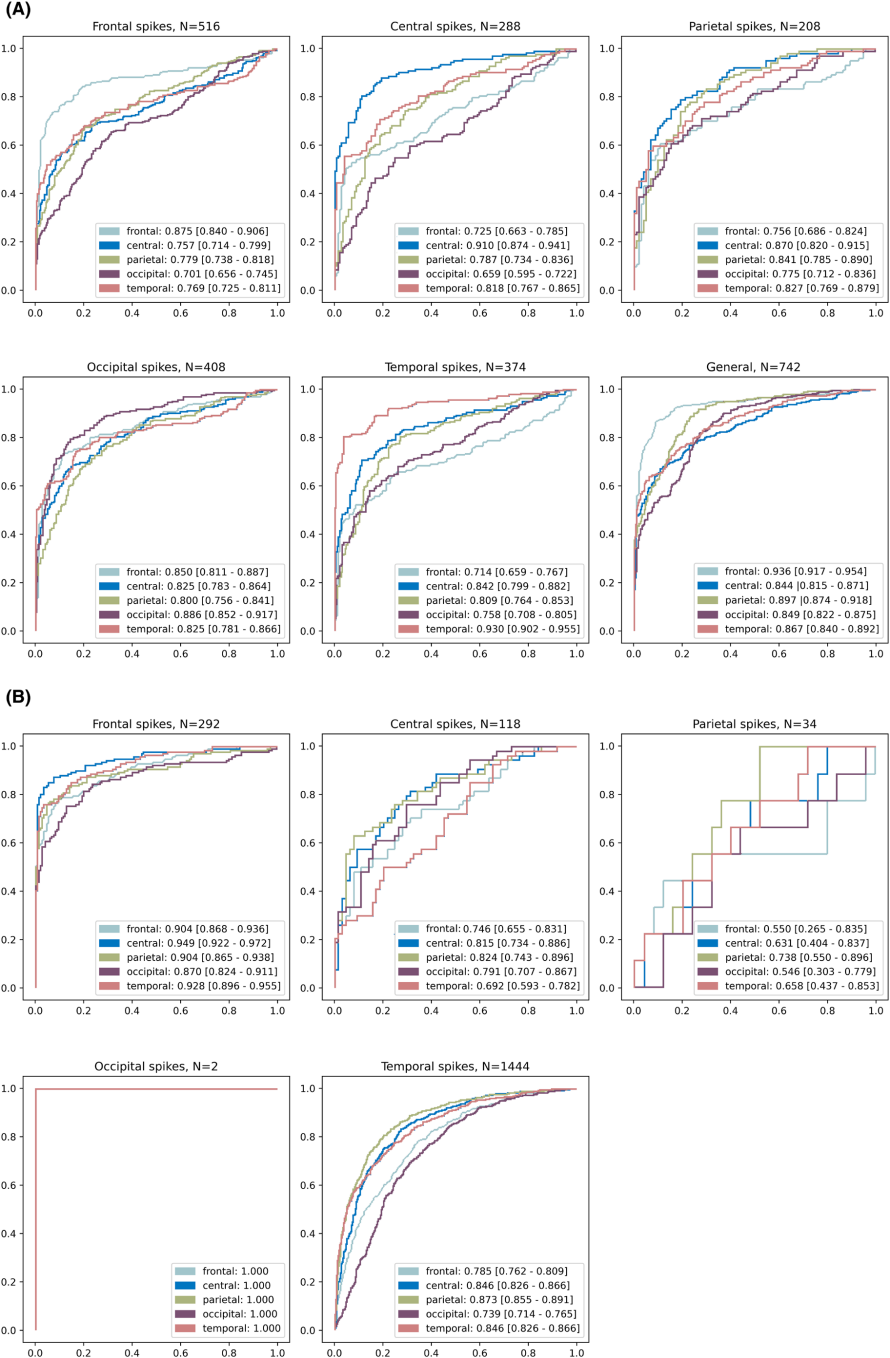
Figure A demonstates the model's performance on the internal dataset for both focal and generalized interictal epileptiform discharges (IEDs). For each type of IED, the model's receiving operator curve was evaluated using two channels from each location: frontal (Fp1, Fp2), central (C3, C4), parietal (P3, P4), occipital (O1, O2), and temporal (T3, T4). Overall, the model performed best when both spike and channel locations aligned. These results generalized to other datasets, as shown in panel B. Due to the low number of samples, results for parietal and occipital spikes in the external dataset were considered not conclusive and not used for any calculations. The presence of only two samples for occipital spikes furthermore explains the area under the receiver operating characteristic curve of 1.

Our final analysis confirmed that Cyclops performs best when both spike and channel locations align. In most cases, the model performs well, even if they do not align. These results were confirmed on an external dataset (Figure 2).

Additional explanations and evaluations can be found in the supplement: Figure S1, flow diagram illustrating the evaluation pipeline of the study; Figure S2, screenshot of EEGnet 3.0, Figure S3, visualization of training procedure; Figure S4, montage visualization; Figure S5, receiver operating characteristic and precision‐recall curves; Figure S6, model prediction visualization; Figure S7, relevance heatmaps; Figure S8, rater agreement evaluation; Table S1, overview of dataset; Table S2, overview of comorbidities; Table S3, overview of medications; Table S4, detailed description of internal dataset; Table S5, distribution of EEG samples and patients by IED location and category; and Table S6, various test metrics for different channel setups.

## 4 DISCUSSION

Cyclops appears to be able to identify most IEDs, irrespective of the number and location of EEG channels available, even with a single channel. *M2* performs almost as well as the full setup, as it covers the regions with the best signal‐to‐noise ratios. To our knowledge, this is the first detailed investigation of this issue. This advance may extend epilepsy monitoring and diagnosis to wider patient populations, reducing the need for complex setups and specialized expertise.

The model's performance is influenced by the data split used for training and testing, details of which can be found in the supplement.

Multiple prior studies have explored the use of reduced‐channel EEG for detecting seizures or closely related patterns.^3^ Studies performed on existing wearable reduced‐channel EEG devices have typically focused on seizure detection^7^, ^8^, ^10^ or sleep staging.^9^ Although multiple studies have trained machine learning models to detect IEDs,^4^ and some outperform human experts,^6^, ^11^, ^12^, ^13^ these studies relied on full‐montage (10–20) EEG. Although some algorithms detect IEDs by independently analyzing each EEG channel,^14^ no prior studies have systematically investigated the effect of reducing EEG channels on the ability of machine learning models to detect IEDs. The novelty of our present work lies not in Cyclops being a convolutional neural network model, but in the finding that even with drastically reduced montages, sufficient information remains to allow clinically useful detection of epileptiform discharges. Future work should explore more advanced model architectures and directly test performance on data recorded on specific wearable devices.

### 4.1 Limitations

Cyclops does not detect all patterns that might be of interest. Nevertheless, IEDs are the primary finding of interest in routine EEGs. External validation was performed with data from other hospitals, but the external validation dataset, although diverse, was relatively small and included neither data from commercial products nor data recorded at locations other than North America. Although Cyclops' excellent performance suggests it can distinguish artifacts from IEDs, we anticipate that real‐world deployment will benefit from further pairing Cyclops with a dedicated artifact‐reduction algorithm.

## 5 CONCLUSIONS

Cyclops is able to accurately detect IEDs using fewer electrodes. This opens the path to diagnostic testing for epilepsy using simpler EEG devices, requiring less expertise for setup.

## CONFLICTS OF INTEREST

M.B.W. was supported by grants from the National Institutes of Health (NIH; RF1AG064312, RF1NS120947, R01AG073410, R01HL161253, R01NS126282, R01AG073598, R01NS131347, R01NS130119) and NSF (2014431). He is a cofounder of and scientific advisor and consultant to Beacon Biosignals and has a personal equity interest in the company. D.G. was supported by NIH K23NS124656. J.J.H. is supported by the VA ORD (I01HX003107‐01A2) and the Human Epilepsy Project. M.G. is supported by the SC BIDS4Health training grant from the NIH (T15LM013977). D.D. is on the scientific advisory board for Beacon Biosignals. E.G. was supported by awards UL1TR002378 and KL2TR002381 (NCATS/NIH) as well as R01NS110347 (NINDS/NIH). J.Y.Y. is supported by the NIH 1UH3NS109557‐01A1 and receives royalty from Elsevier. M.G. is supported by funding from UNEEG.

## ETHICS STATEMENT

We confirm that we have read the Journal's position on issues involved in ethical publication and affirm that this report is consistent with those guidelines.

## AUTHOR CONTRIBUTIONS

*Conceptualization:* M. Brandon Westover and Robert Thomas. *Data curation:* Moritz Alkofer, Wolfgang Ganglberger, Jules Beal, Manu Hegde, Joon‐Yi Kang, Ji Yeoun Yoo, Mattia Galanti, Liu Lin Thio, Ekrem Kutluay, Zeke Campbell, Sarah Schmitt, Ezequiel Gleichgerrcht, Elizabeth Waterhouse, Stephan Eisenschenk, Michael A. Gelfand, Rani K. Singh, Kristin E. Wills, Erik‐Jan Meulenbrugge, Brian Dean, Jonathan J. Halford, Daniel Goldenholz, Jin Jing, and M. Brandon Westover. *Formal analysis:* Moritz Alkofer (experiments and validations). *Funding acquisition:* M. Brandon Westover. *Investigation:* Wolfgang Ganglberger, Jules Beal, Manu Hegde, Joon‐Yi Kang, Ji Yeoun Yoo, Mattia Galanti, Liu Lin Thio, Ekrem Kutluay, Zeke Campbell, Sarah Schmitt, Ezequiel Gleichgerrcht, Elizabeth Waterhouse, Maria R. Lopez, Stephan Eisenschenk, Michael A. Gelfand, Rani K. Singh, Kristin E. Wills, Erik‐Jan Meulenbrugge, Dennis Dlugos, Brian Dean, Jonathan J. Halford, Daniel Goldenholz, Jin Jing, and M. Brandon Westover. *Methodology:* M. Brandon Westover and Moritz Alkofer. *Project administration:* Moritz Alkofer. *Resources:* M. Brandon Westover and Robert Thomas. *Software:* Moritz Alkofer and Chaoqi Yang. *Supervision:* M. Brandon Westover, Robert Thomas, and Daniel Goldenholz. *Validation:* Moritz Alkofer. *Visualization:* Moritz Alkofer. *Writing—original draft preparation:* Moritz Alkofer, M. Brandon Westover, Daniel Goldenholz, and Wolfgang Ganglberger. *Writing—review and editing:* Moritz Alkofer, Chaoqi Yang, Wolfgang Ganglberger, Jules Beal, Manu Hegde, Joon‐Yi Kang, Ji Yeoun Yoo, Mattia Galanti, Liu Lin Thio, Ekrem Kutluay, Zeke Campbell, Sarah Schmitt, Ezequiel Gleichgerrcht, Elizabeth Waterhouse, Maria R. Lopez, Stephan Eisenschenk, Michael A. Gelfand, Rani K. Singh, Kristin E. Wills, Erik‐Jan Meulenbrugge, Dennis Dlugos, Brian Dean, Jonathan J. Halford, Daniel Goldenholz, Jin Jing, and M. Brandon Westover.

## Supporting information


Data S1.

Data S2.

## Data Availability

The internal data that support the findings of this study are openly available at https://bdsp.io/content/cyclops/2.0, an NIH‐approved data sharing mechanism. The Human Epilepsy Project data, used for external validation, are available on request from the investigators who lead that project. Code available at https://github.com/bdsp‐core/cyclops.
