# Supplementary material for: Automated detection of interictal epileptiform discharges with few electroencephalographic channels
Source: Epilepsia. 2025 May 3;66(7):e114–20. doi: 10.1111/epi.18431 (PMC12291011; doi:10.1111/epi.18431)
Supplement: Supplementary file 1 — Data S1. [file EPI-66-e114-s001.docx]

**Supplements**

**Sections:**

Model

Detailed dataset information

Dataset split description

Training details

Results

References

**Tables:**

Table S1) Overview dataset

Table S2) Overview comorbidities

Table S3) Overview medications

Table S4) Detailed description of internal dataset

Table S5) Distribution of EEG samples and patients by IED location and category

Table S6) Various test metrics for different channel setups

**Figures:**

Figure S1) Flow diagram illustrating the evaluation pipeline of the study.

Figure S2) Screenshot of EEGNet 3.0

Figure S3) Visualization of training procedure

Figure S4) Montage visualization

Figure S5) ROC and PRC curves

Figure S6) Model prediction visualization

Figure S7) Relevance heatmaps

Figure S8) Rater agreement evaluation

**Figure S1)**

Flow diagram illustrating the evaluation pipeline of the study. All supplementary analysis was performed using the primary split test set and model A.

**Model**

The model called ‘Cyclops’ is a convolutional neural network (CNN) with 364,000 parameters, developed by Jing et al^1^. It was inspired by Hannun et al^2^, who developed it for single channel echocardiogram analysis. The architecture consists of 6 blocks and has an input shape of [128 timesteps, 19 signal channels, and 1 feature channel], with 19 channels prepared in the average montage and 128 timesteps, representing a 1s snippet recorded at 128 Hz.

The first block applies a temporal convolution for each signal channel, creating 32 feature channels. The second block applies a channel-wise convolution to all signal channels, collating them into one, followed by a temporal convolution. The following blocks 3-6 alternate between downsampling the number of timesteps by a factor of 2 and increasing the number of feature channels by 32. Each block applies batch norm, followed by a leaky ReLU activation function and a dropout layer with p = 0.2. The output is flattened to a vector of length 256 and fed to a dense layer with a sigmoid activation function.

**Dataset**

The datasets used in this study consisted of EEG recordings acquired using the 21 electrodes of the International 10-20 System^3^. Spikes were identified based on expert consensus, which is most closely aligned with the informal 1999 definition^4^ (as opposed to the more recently proposed 2020 IFCN^5^ criteria).

The 10-20 System has many advantages, including greater redundancy in case a signal is lost from one electrode, greater flexibility in waveform visualization (re-montaging), and greater input information to aid human artifact recognition and automated artifact reduction. But placement of the 10-20 System electrode array takes a long time to set up and is uncomfortable for the patient, especially due to the use of EEG gel. Using fewer and/or dry electrodes in wearable EEG decreases setup cost and discomfort, increases portability and thus makes long term recordings easier to obtain.

| **Group name** | **Patients** | **Recordings** | **Female** | **Samples** | **Spikes** | **Routine** | **LTM** | **EMU** | **Other** |
| --- | --- | --- | --- | --- | --- | --- | --- | --- | --- |
| Total | 3378 | 3631 | 1458 (48) | 115454 | 62860 (54) | 2110 (57) | 998 (27) | 199 (5) | 324 (9) |
| Internal | 3327 | 3580 | 1458 (48) | 112154 | 61210 (55) | 2110 (59) | 998 (28) | 199 (6) | 273 (8) |
| External | 51 | 51 | ? | 3300 | 1650 (50) | ? | ? | ? | ? |
| 0 to < 5 | 177 | 207 | 80 (45) | 10100 | 6926 (69) | 140 (68) | 57 (28) | 10 (5) | 0 (0) |
| 5 to < 13 | 323 | 400 | 142 (44) | 25835 | 20887 (81) | 315 (79) | 52 (13) | 33 (8) | 0 (0) |
| 13 to < 18 | 178 | 220 | 78 (44) | 11189 | 8344 (75) | 171 (78) | 31 (14) | 17 (8) | 1 (0) |
| 18 to < 30 | 392 | 425 | 179 (46) | 11641 | 6229 (54) | 280 (66) | 78 (18) | 64 (15) | 3 (1) |
| 30 to < 50 | 471 | 489 | 230 (49) | 10307 | 3585 (35) | 298 (61) | 147 (30) | 44 (9) | 0 (0) |
| 50 to < 65 | 599 | 620 | 283 (47) | 12471 | 4065 (33) | 356 (56) | 239 (39) | 24 (4) | 1 (0) |
| 65 to < 75 | 442 | 455 | 206 (47) | 10404 | 3831 (37) | 262 (57) | 186 (41) | 5 (1) | 2 (0) |
| 75 to < inf | 487 | 500 | 260 (53) | 10961 | 3595 (33) | 288 (57) | 208 (42) | 2 (0) | 2 (0) |
| Unknown | 309 | 315 | 0 (0) | 12546 | 5398 (43) | 0 (0) | 0 (0) | 0 (0) | 315 (100) |

**Table S1: Overview of study samples**

Abbreviations: LTM, long-term monitoring; EMU, epilepsy monitoring unit. In the rare case of having multiple recordings per patient, the age is assigned based on the date of the first recording. In this table, a sample is considered to have a spike if over 50% of the experts classified it as such. A description of the underlying datasets is provided in Table S3a-c.

| Category | Percentage |
| --- | --- |
| Headache Disorders | 36.55% |
| Seizure Disorders | 34.3% |
| Sleep Disorders | 22.90% |
| Movement And Cerebellar Disorders | 18.67% |
| Cerebral Lobe Dysfunction | 17.04% |
| Other Neurological Disorders | 15.87% |
| Developmental Delay | 10.43% |
| Peripheral Nervous System Disorders | 8.63% |
| Intracranial And Spinal Tumors | 7.97% |
| Cerebral Degeneration | 7.15% |
| Infections | 6.16% |
| Behavioral/Cognitive Syndromes | 5.50% |
| Muscular Dystrophies And Other Myopathies | 2.86% |
| Cranial Nerve Disorders | 0.66% |
| Motor Neuron Diseases | 0.30% |
| Genetic Disorders | 0.03% |
| Cerebrovascular Diseases | 0% |
| Miscellaneous | 93.42% |

Table S2) Comorbidities

List of neurological comorbidities and their prevalence in the study population. Classification based on ICD codes for neurology^6^.

| **Medication Class** | **Percentage** |
| --- | --- |
| Neurological | 21.45% |
| Psychiatric | 16.20% |
| Cardiovascular | 14.77% |
| Gastrointestinal | 12.01% |
| Ophthalmic and otolaryngological | 5.96% |
| Endocrinological | 5.94% |
| Respiratory | 4.66% |
| Infectious diseases | 4.30% |
| Renal | 2.90% |
| Urological | 0.89% |
| Rheumatologic | 0.58% |
| Dermatological | 0.02% |

**Table S3) Medication**

List of medication classes and their prescription in the study population. Classification based on electronic health records.

**Detailed Dataset information**

The internal dataset described in Table S1 consisted of three datasets described in Table S3 a-c. Internal-A contains samples annotated for IED presence and location by 3-23 (median 8) independent neurologists who specialize in epilepsy and clinical neurophysiology (from here on called “experts”). Internal-B contained IED samples that were labeled indirectly (“pseudolabeled”) by their morphological similarity to spikes with expert labels, as detailed in^7^. Internal-C contained no IEDs (e.g. negative samples). For internal A-C the reference electrode was placed at C2 (second cervical vertebrae).

The external dataset contained labels for IED presence and location from three experts. As described in the section on dataset splits, only data evaluated by multiple experts was used for testing.

| **Group name** | **Patients** | **Recordings** | **Female** | **Samples** | **Spikes** | **Routine** | **LTM** | **EMU** | **Other unit** |
| --- | --- | --- | --- | --- | --- | --- | --- | --- | --- |
| Total | 1892 | 2120 | 855 (50) | 18804 | 10671 (56) | 1017 (48) | 741 (35) | 166 (8) | 196 (9) |
| 0 to < 5 | 109 | 138 | 48 (44) | 1289 | 930 (72) | 79 (56) | 49 (36) | 10 (7) | 0 (0) |
| 5 to < 13 | 256 | 332 | 117 (46) | 3595 | 2781 (77) | 253 (76) | 49 (15) | 30 (9) | 0 (0) |
| 13 to < 18 | 132 | 173 | 60 (45) | 1630 | 1220 (75) | 129 (75) | 28 (16) | 16 (9) | 0 (0) |
| 18 to < 30 | 213 | 243 | 107 (50) | 1480 | 1017 (69) | 127 (52) | 56 (23) | 58 (24) | 2 (1) |
| 30 to < 50 | 212 | 228 | 109 (51) | 1072 | 625 (57) | 102 (45) | 92 (40) | 34 (15) | 0 (0) |
| 50 to < 65 | 295 | 307 | 153 (52) | 1291 | 681 (53) | 126 (41) | 166 (54) | 14 (5) | 1 (0) |
| 65 to < 75 | 235 | 243 | 121 (51) | 1245 | 640 (51) | 102 (42) | 137 (56) | 3 (1) | 1 (0) |
| 75 to < inf | 254 | 264 | 140 (55) | 1256 | 586 (47) | 99 (38) | 164 (62) | 1 (0) | 0 (0) |
| Unknown | 186 | 192 | 0 (0) | 5946 | 2191 (37) | 0 (0) | 0 (0) | 0 (0) | 192 (100) |

Table S4a: Internal A encompassed 18804 EEG samples from 2120 patients and was based on the dataset derived in[13](https://www.zotero.org/google-docs/?0iDxiA). These samples were annotated with binary labels by 3 to 23 (median 8) clinical experts to indicate the presence of IEDs. To reflect the consensus among neurologists with clinical electroencephalography fellowship training, these binary labels were aggregated into a soft ‘consensus’ label, quantifying the fraction of experts agreeing on IED presence. A subset of 1,268 positive samples were further classified based on their localization by one expert (MBW), as shown in Table S5.

|  | **Patients** | **Recordings** | **Female** | **Samples** | **Spikes** | **Routine** | **LTM** | **EMU** | **Other unit** |
| --- | --- | --- | --- | --- | --- | --- | --- | --- | --- |
| Total | 625 | 749 | 316 (52) | 63350 | 50539 (80) | 521 (70) | 158 (21) | 48 (6) | 22 (3) |
| 0 to < 5 | 55 | 73 | 23 (42) | 7424 | 5996 (81) | 57 (78) | 14 (19) | 2 (3) | 0 (0) |
| 5 to < 13 | 156 | 198 | 79 (51) | 20865 | 18106 (87) | 168 (85) | 18 (9) | 12 (6) | 0 (0) |
| 13 to < 18 | 75 | 101 | 35 (47) | 8588 | 7124 (83) | 83 (82) | 9 (9) | 9 (9) | 0 (0) |
| 18 to < 30 | 84 | 100 | 47 (56) | 6416 | 5212 (81) | 68 (68) | 15 (15) | 16 (16) | 1 (1) |
| 30 to < 50 | 58 | 66 | 31 (53) | 3957 | 2960 (75) | 48 (73) | 10 (15) | 8 (12) | 0 (0) |
| 50 to < 65 | 59 | 65 | 28 (47) | 4663 | 3384 (73) | 36 (55) | 28 (43) | 1 (2) | 0 (0) |
| 65 to < 75 | 52 | 53 | 34 (65) | 4809 | 3191 (66) | 27 (51) | 26 (49) | 0 (0) | 0 (0) |
| 75 to < inf | 70 | 72 | 39 (56) | 4814 | 3009 (63) | 34 (47) | 38 (53) | 0 (0) | 0 (0) |
| Unknown | 16 | 21 | ? | 1814 | 1557 (86) | ? | ? | ? | ? |

Table S4b): Internal B comprised 63,350 samples from 625 patients, each identified by one expert. Soft labels were assigned based on morphological similarity (determined by dynamic time warping) with directly labeled samples from Internal-A. This approach, as explained in[4](https://www.zotero.org/google-docs/?oXch0i), added 50539 positive samples.

|  | Patients | Recordings | Female | Samples | Spikes | Routine | LTM | EMU | Other unit |
| --- | --- | --- | --- | --- | --- | --- | --- | --- | --- |
| Total | 1508 | 1513 | 636 (44) | 30000 | 0 (0) | 1114 (74) | 283 (19) | 37 (2) | 79 (5) |
| 0 to < 5 | 70 | 70 | 33 (47) | 1387 | 0 (0) | 62 (89) | 8 (11) | 0 (0) | 0 (0) |
| 5 to < 13 | 69 | 69 | 26 (38) | 1375 | 0 (0) | 62 (90) | 4 (6) | 3 (4) | 0 (0) |
| 13 to < 18 | 49 | 49 | 19 (39) | 971 | 0 (0) | 43 (88) | 4 (8) | 1 (2) | 1 (2) |
| 18 to < 30 | 189 | 189 | 76 (40) | 3745 | 0 (0) | 154 (81) | 26 (14) | 8 (4) | 1 (1) |
| 30 to < 50 | 267 | 267 | 124 (46) | 5278 | 0 (0) | 197 (74) | 59 (22) | 11 (4) | 0 (0) |
| 50 to < 65 | 327 | 329 | 142 (43) | 6517 | 0 (0) | 238 (72) | 80 (24) | 11 (3) | 0 (0) |
| 65 to < 75 | 218 | 220 | 90 (41) | 4350 | 0 (0) | 165 (75) | 52 (24) | 2 (1) | 1 (0) |
| 75 to < inf | 245 | 246 | 126 (51) | 4891 | 0 (0) | 193 (78) | 50 (20) | 1 (0) | 2 (1) |
| Unknown | 74 | 74 | ? | 1486 | 0 (0) | ? | ? | ? | ? |

Table S4c: Internal C included 30,000 negative samples from 1434 patients. These samples were randomly selected from continuous EEGs without any reported IEDs and were used to balance Internal-A and -B.

The **external dataset** was derived from the EEG recordings of the Human Epilepsy Project (HEP), a 6-year, prospective, observational, 29-site multicenter study, setup to evaluate clinical, electrographic, and neuroimaging characteristics of patients with newly-diagnosed focal epilepsy. Patients were 18–60 years old with documented focal epilepsy who had been started on an anti-seizure medicine within 4 months from the time of enrollment. The diagnosis was confirmed by the HEP Semiology Core. The study protocol specified that each subject was to receive one sleep-deprived EEG recording of at least 2 hours in length. Out of 488 subjects enrolled, some type of EEG was collected for 448 (for some of these subjects, only screen capture of a few pages of EEG was sent), 440 subjects had digital EEG data collected at the study site, and 327 subjects had EEG data submitted from the study site to the HEP Core Committee in one of the many file formats of a neurophysiology original equipment manufacturer (OEM). Central HEP staff converted the EEG data from the OEM format (some of which were proprietary formats) into Persyst .lay/.dat format using Persyst software. These Persyst .lay files were then inputted into the EEGlab environment. EEGLab is an interactive MATLAB toolbox for processing continuous and event-related EEG^8^. Using EEGLab, EEG datasets were then (1) converted to EEGLab .set files, (2) periods of EEG consisting of noise (without useable EEG signal) at the beginning and the end of the files were removed by manual inspection by JJH, (3) the best ECG channel was selected and other ECG channels were discarded, and (4) only 10-20 and 10-10 channels were included that were present in at least 10 of the HEP EEG datasets (and channels were assigned standard names) such that a single EEGLab .locs montage file could be used for all of HEP subject’s .set files.

Several sets of EDF files were created from the .set files described above, and the set of EDF files used for this study consisted of all 10-20 montage EEG channels plus one channel of ECG.

The length of the 327 EEG recordings collected was 14s - 64 hr with median and mean length 1.24 hr and 3 hr 2 min, respectively. A total of 57% (188/327) of the EEG recordings were longer than one hour. Three experts (assigned randomly and evenly from a pool of 10 experts participating in EEG annotation) marked within these recordings a one-hour EEG epoch (containing all channels) of the first hour of sleep, or an hour with as much sleep as possible if they could not find an hour of sleep. Annotation of HEP EEG data was performed using a new iteration of the EEGnet webserver annotation system^9,10^. The graphic user interface (GUI) of EEGnet 3.0 is shown in Figure S1.

Each of the one-hour EEG epochs was annotated using EEGnet by 3 experts (randomly and evenly drawn from a pool of 13 experts), who marked the location of every IED in each recording. Experts were instructed to label the time point of the peak of the IED in the channel in which they thought it was best represented, using any of seven available montages. Experts could control global and/or individual channel gain, filtering (high pass, low pass, notch), and montaging. A total of 7903 IEDs were marked by the experts in 188 subjects across 20 different clinical sites, with 3 experts randomly and evenly assigned to each subject EEG. The range of IED annotations marked in those subjects who had at least one IED marked in their recording (96 EEGs) was 1 - 1471. HEP1 EEGs which are less than one hour of duration were not used in this study because labeling of IEDs by HEP investigators using EEGnet has not yet been completed.

The annotated IEDs that occurred within 0.1 sec of each other were clustered and considered one IED if at least 2 experts (out of 3) marked it. The clustering algorithm was designed to group IEDs based on time proximity and expert identity. The algorithm analyzes sequences of four consecutive expert-labeled IEDs at a time (belonging to the same subject), checking the time intervals between each of four expert-labeled IEDs against a set time threshold (DT = 0.1 sec). If consecutive markings are made by the same expert, the sequence is advanced by one IED. Clusters are formed if the time intervals between markings fall below DT. Subsequent markings are included if they occur less than DT after the final marker and were annotated by an independent expert. This iterative process ensures accurate grouping of IEDs, taking into account both the timing and the independence of expert judgments. A total of 1650 IED clusters were produced. From all subjects who had at least one IED labeled in their EEG, 3300 (both containing IEDs and not) 10-second samples (including all EEG channels) were obtained. The range of IED annotations marked per subject after clustering was 1-309 with a median of 6.0 and a mean of 32.3 respectively. While some patients contribute a high number of samples, the majority has relatively few, indicating no major oversampling problem. All EEG recordings were sampled at a rate of at least 200 Hz (sampling rate range 200-1024 Hz, median: 256 Hz) and recorded using the Standard 10-20 montage. Spikes in positive samples were temporally centered. Preprocessing was performed by first downsampling to 128 Hz then applying a 60 Hz notch filter, followed by a zero-lag 0.5-60 Hz band-pass filter.

To infer the approximate brain region associated with each spike, channels were mapped to broad anatomical locations based on the following channel-location pairings: frontal (Fp1, Fp2), central (C3, Cz, C4), parietal (P3, P4), occipital (O1, O2), and temporal (T3, T4). An IED was considered to be located on a specific channel if at least two experts agreed on the channel. Ten samples were mapped to two locations, because two or more raters located the IED on each of two different bipolar channels. The resulting mapping is shown in Table S5b.

**Figure S2)** Screenshot of EEGNet 3.0.

| **Location** | **Samples** | **Patients** |
| --- | --- | --- |
| **frontal** | 258 | 43 |
| **central** | 144 | 47 |
| **parietal** | 104 | 31 |
| **occipital** | 204 | 25 |
| **temporal** | 187 | 53 |
| **general** | 371 | 72 |
| **Total** | 1268 | 271 |

**Table S5a:** internal dataset

| **Location** | **Samples** | **Patients** |
| --- | --- | --- |
| **frontal** | 146 | 16 |
| **central** | 59 | 8 |
| **parietal** | 17 | 1 |
| **occipital** | 1 | 1 |
| **temporal** | 722 | 39 |
| **Total** | 934 | 51 |

**Table S5b:** external dataset

**Table S5:** Distribution of external EEG samples and patients by IED location and category.

**Training**

**Figure S3:** The model was trained to predict the percentage of experts voting that a given sample contains an IED. During training, random channels were set to zero, leading to IED representations robust to channel deletion. Data was provided to the model in average montage, calculated using only the non-deleted channels.

**Dataset splits**

To facilitate nuanced analysis, the datasets A, B, and C were combined using two different splits: a “primary split” and a “secondary split”. Each split was designed to assess the model's performance in detecting IEDs, with one focusing on the model’s general performance and the other on the influence of spike and channel location on model performance. To prevent data leakage, both splits were assigned patient wise.

The **primary split** combined dataset A, B, and C. First, the data was patient-wise distributed to train/val/test using an 80/10/10 split. During the testing phase, only positive samples confirmed by at least eight experts, with a consensus of seven or more and unanimously confirmed negative samples were included. This resulted in 97742/ 14420/ 982 samples.

The **secondary split** combined Dataset A, B, and 10,000 samples of dataset C. All 1,263 localized samples and negatives from the same patients were assigned to the test set. For stratification, non localized samples from the same patients were discarded. The remaining data was distributed patient-wise on train/val using an 80/20 split. All the localized spikes from dataset A were used for testing and location wise balanced with spike-free samples from dataset A. This results in 56849/15067/2247 samples.

The stringent requirements for spike presence consensus were relaxed due to the scarcity of samples with localization labels.

**Figure S4: Montages used**

Visualization of all montages used for evaluation. Not used channels are set to zero, not deleted. The data shape remains fixed (19 channels, 128 timesteps) throughout all montages.

**Figure S4a): Clinically inspired montages**

Various montages used to gauge real world performance, including montages similar to those used by two commercial subscalp systems. **M1**: T3, F7, T4, and F8^11^; **M2**: T5, P3, Pz, T6 and P4^12^ as well as reduced montages used by commercially available EEG devices, **M3**: Fp1, Fp2, F7, F8, O1, and O2^13^ and **M4**: Fp1, Fp2, F7, F8, T3, T5, T4, T6, O1, and O2^14^, as well as the channels available in typical clinical polysomnography data, **M-PSG**: F3, C3, O1, F4, C4 and O4 and **all 10-20 channels**: Fp1, F3, C3, P3, F7, T3, T5, O1, Fz, Cz, Pz, Fp2, F4, C4, P4, F8, T4, T6 and O2. Performance for all montages can be viewed in Figure 1 of the main manuscript.

**Figure S4b): randomized montages**Examples for randomized montages with fixed N. Performance for every number of channels can be viewed in Figure 1 of the main manuscript.

**Figure S4c): Focalized montages**

Channels used for assessment performance on focal IED relative to electrode location. Frontal (Fp1, Fp2), central (C3, C4), parietal (P3, P4), occipital (O1, O2), temporal (T3, T4). Performance for each montage can be viewed in Figure 2 of the main manuscript.

**Results**

**Figure S5: Receiver Operating Characteristic Curve and Precision-Recall Curve for various channel configurations.**

The legend displays the AUC values for each configuration. Dots on the curves represent the model's operating threshold, determined by minimizing the root mean squared error on the binarized labels on the validation set. All configurations demonstrate exceptional discrimination abilities.

| Channels | Accuracy | Sensitivity | Specificity | Precision | Operating_point |
| --- | --- | --- | --- | --- | --- |
| M1 | 0.827 [0.802-0.850] (92.8) | 0.811 [0.775-0.845] (97.6) | 0.843 [0.810-0.875] (88.7) | 0.838 [0.803-0.870] (88.7) | 0.52 |
| M2 | 0.893 [0.874-0.912] (100.2)* | 0.857 [0.827-0.888] (103.2)* | 0.929 [0.905-0.951] (97.7) | 0.923 [0.898-0.947] (97.8) | 0.54 |
| M3 | 0.851 [0.829-0.873] (95.6) | 0.768 [0.730-0.805] (92.4) | 0.935 [0.912-0.956] (98.3) | 0.922 [0.895-0.946] (97.6) | 0.65 |
| M4 | 0.881 [0.860-0.901] (98.9) | 0.821 [0.787-0.855] (98.8) | 0.941 [0.919-0.961] (99.0) | 0.933 [0.908-0.955] (98.8) | 0.63 |
| M-PSG | 0.864 [0.842-0.885] (97.0) | 0.793 [0.756-0.827] (95.4) | 0.935 [0.912-0.956] (98.3) | 0.924 [0.898-0.948] (97.9) | 0.6 |
| all 10-20 channels | 0.891 [0.871-0.909] (100.0) | 0.831 [0.797-0.863] (100.0) | 0.951 [0.931-0.969] (100.0) | 0.944 [0.921-0.965] (100.0) | 0.57 |

**Table S6: Evaluation of operating points for different channels setups using various test metrics.**

Square brackets denote the 95% confidence intervals, parentheses the performance as a percentage of the full 10-20 channel setup. *note: The M2 montage supersedes the full setup in accuracy and sensitivity, but has reduced specificity and precision.

**Figure S6:** Visual inspection of the test prediction spectrum for the M4 setup (Fp1, Fp2, F7, F8, T3, T5, T4, T6, O1, O2) trained on the primary split. The value above each column shows the model's prediction. The red bar represents an amplitude of 100 microvolts. The model only sees the central second. **A:** Cyclops’ predictions on positive samples. False negatives, where the model incorrectly predicted normal readings, are positioned on the left. True positives, where the model correctly detected abnormalities, are on the right. **B)** Cyclops’ predictions on negative samples. True negatives, correctly classified as normal, are shown on the left. False positives, incorrectly flagged as abnormal, are on the right.

**Figure S7: Relevance heatmaps for EEG with and without IEDs.**

The model emphasizes spikes and sharp EEG instead of spurious correlations. 1s model input window in color and 2s context in Gray. The heatmap was created using layerwise relevance propagation and software for^15^.

**Figure S8: Cyclops evaluation for a range of n random channels.**

The rater agreement shows the lower bound of agreement for a sample to be considered a spike. For the different rater agreements the AUCs are 8/8: 0.886; 7/8: 0.866; 6/8: 0.848; 5/8: 0.834; 4/8: 0.816.

**Supplemental References**

1. Jing J, Sun H, Kim JA, et al. Development of Expert-Level Automated Detection of Epileptiform Discharges During Electroencephalogram Interpretation. *JAMA Neurol*. 2020;77(1):103-108. doi:10.1001/jamaneurol.2019.3485

2. Hannun AY, Rajpurkar P, Haghpanahi M, et al. Cardiologist-Level Arrhythmia Detection and Classification in Ambulatory Electrocardiograms Using a Deep Neural Network. *Nat Med*. 2019;25(1):65-69. doi:10.1038/s41591-018-0268-3

3. Klem GH, Lüders HO, Jasper HH, Elger C. The ten-twenty electrode system of the International Federation. The International Federation of Clinical Neurophysiology. *Electroencephalogr Clin Neurophysiol Suppl*. 1999;52:3-6.

4. G. Deuschl, A. Eisen. Recommendations for the practice of clinical neurophysiology: guidelines of the International Federation of Clinical Neurophysiology. *Electroencephalogr Clin Neurophysiol Suppl*. 1999;52:1-304.

5. Kural MA, Duez L, Sejer Hansen V, et al. Criteria for defining interictal epileptiform discharges in EEG: A clinical validation study. *Neurology*. 2020;94(20). doi:10.1212/WNL.0000000000009439

6. Quest Diagnostics. ICD-10-CM Codes for Neurology. January 10, 2015. Accessed February 20, 2025. https://www.questdiagnostics.com/content/dam/corporate/restricted/documents/icd-10-codes/cpt-2015/icd_10_codes_for_neurology-mi4957/ICD_10_Codes_for_Neurology-MI4957.pdf

7. Jing J, Dauwels J, Rakthanmanon T, Keogh E, Cash SS, Westover MB. Rapid Annotation of Interictal Epileptiform Discharges via Template Matching under Dynamic Time Warping. *J Neurosci Methods*. 2016;274:179-190. doi:10.1016/j.jneumeth.2016.02.025

8. Delorme A, Makeig S. EEGLAB: an open source toolbox for analysis of single-trial EEG dynamics including independent component analysis. *J Neurosci Methods*. 2004;134(1):9-21. doi:10.1016/j.jneumeth.2003.10.009

9. Halford JJ, Schalkoff RJ, Zhou J, et al. Standardized database development for EEG epileptiform transient detection: EEGnet scoring system and machine learning analysis. *J Neurosci Methods*. 2013;212(2):308-316. doi:10.1016/j.jneumeth.2012.11.005

10. F Battaglia, M Galanti, Gugliandolo G, et al. Neurophysiology Signal Codecs for the DICOM® Standard: Preliminary Results. *IEEE International Symposium on Medical Measurements and Applications (MeMeA)*. Published online 2024.

11. Duun-Henriksen J, Baud M, Richardson MP, et al. A new era in electroencephalographic monitoring? Subscalp devices for ultra–long-term recordings. *Epilepsia*. 2020;61(9):1805-1817. doi:10.1111/epi.16630

12. Stirling RE, Maturana MI, Karoly PJ, et al. Seizure Forecasting Using a Novel Sub-Scalp Ultra-Long Term EEG Monitoring System. *Front Neurol*. 2021;12:713794. doi:10.3389/fneur.2021.713794

13. Arnal PJ, Thorey V, Debellemaniere E, et al. The Dreem Headband compared to polysomnography for electroencephalographic signal acquisition and sleep staging. *Sleep*. 2020;43(11):zsaa097. doi:10.1093/sleep/zsaa097

14. Vespa PM, Olson DM, John S, et al. Evaluating the Clinical Impact of Rapid Response Electroencephalography: The DECIDE Multicenter Prospective Observational Clinical Study. *Crit Care Med*. 2020;48(9):1249-1257. doi:10.1097/CCM.0000000000004428

15. Anders CJ, Neumann D, Samek W, Müller KR, Lapuschkin S. Software for Dataset-wide XAI: From Local Explanations to Global Insights with Zennit, CoRelAy, and ViRelAy. Published online February 28, 2023. doi:10.48550/arXiv.2106.13200
