# Supplementary figures and images for: Automated detection of interictal epileptiform discharges with few electroencephalographic channels
Source: Epilepsia. 2025 May 3;66(7):e114–20. doi: 10.1111/epi.18431 (PMC12291011; doi:10.1111/epi.18431)

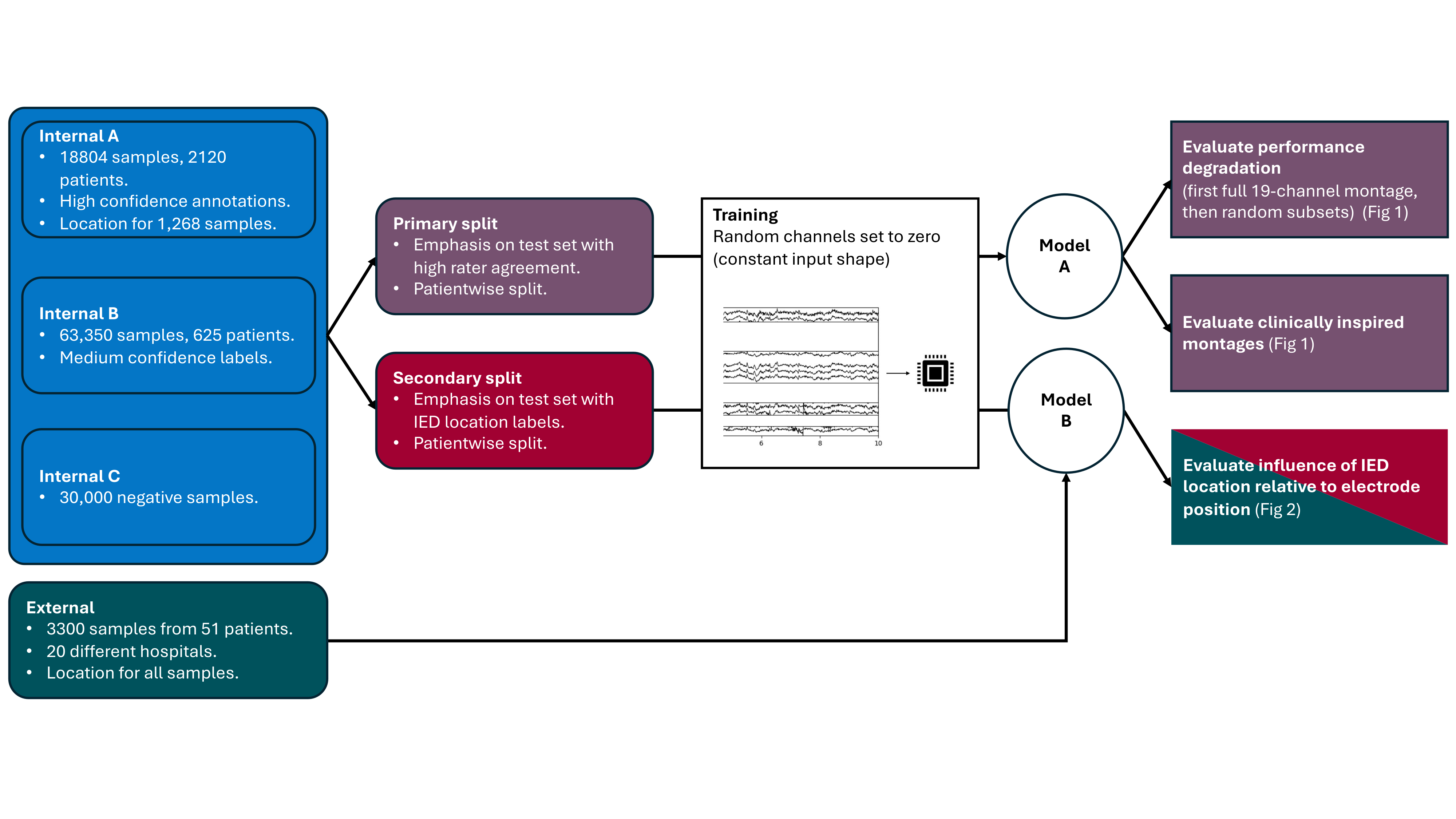

Supplement: Supplementary file 2 — Data S2. [file EPI-66-e114-s002.zip › EPI_18431_f1_Alkofer-FigS1.tiff]

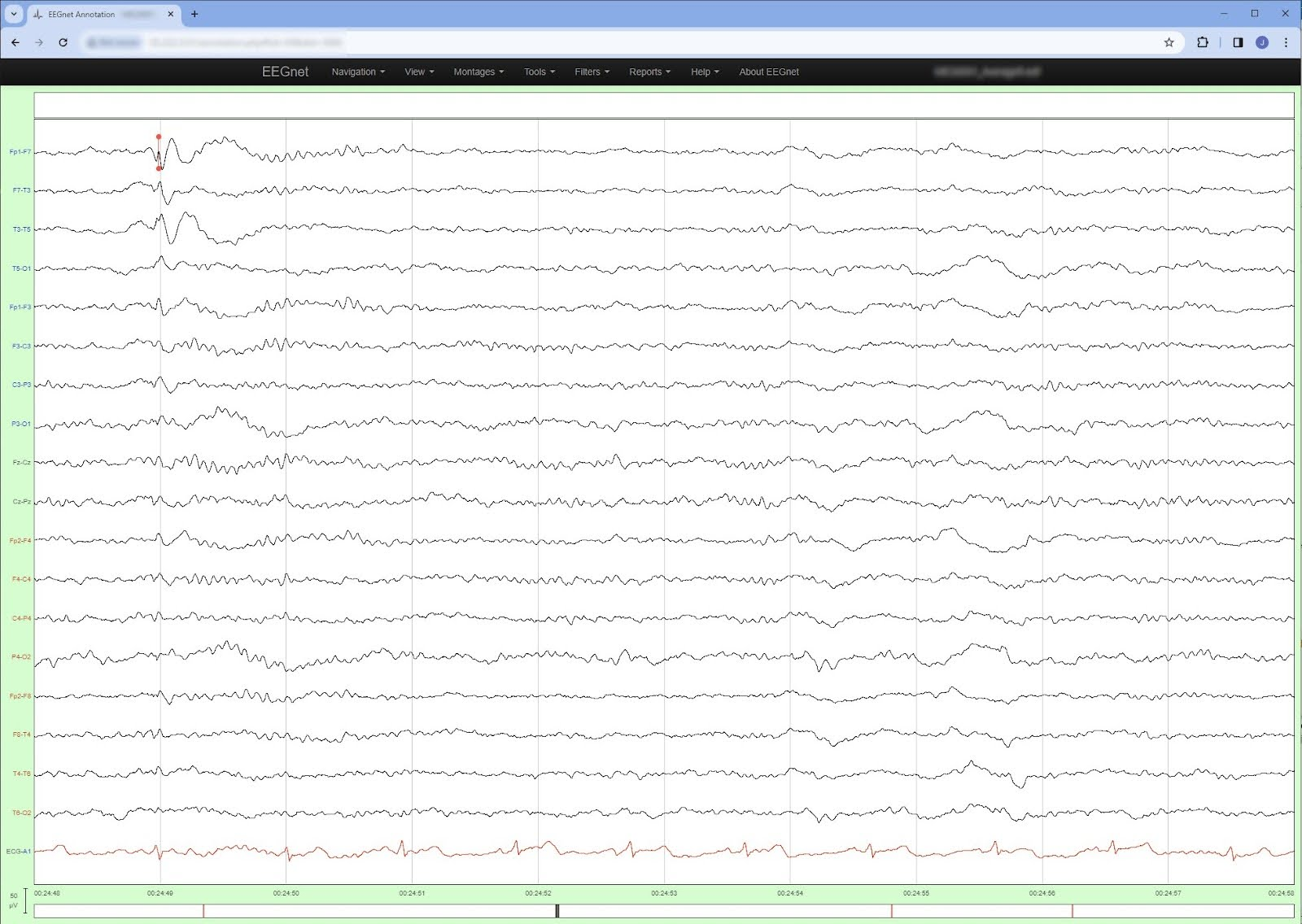

Supplement: Supplementary file 2 — Data S2. [file EPI-66-e114-s002.zip › EPI_18431_f2_Alkofer-FigS2.tiff]

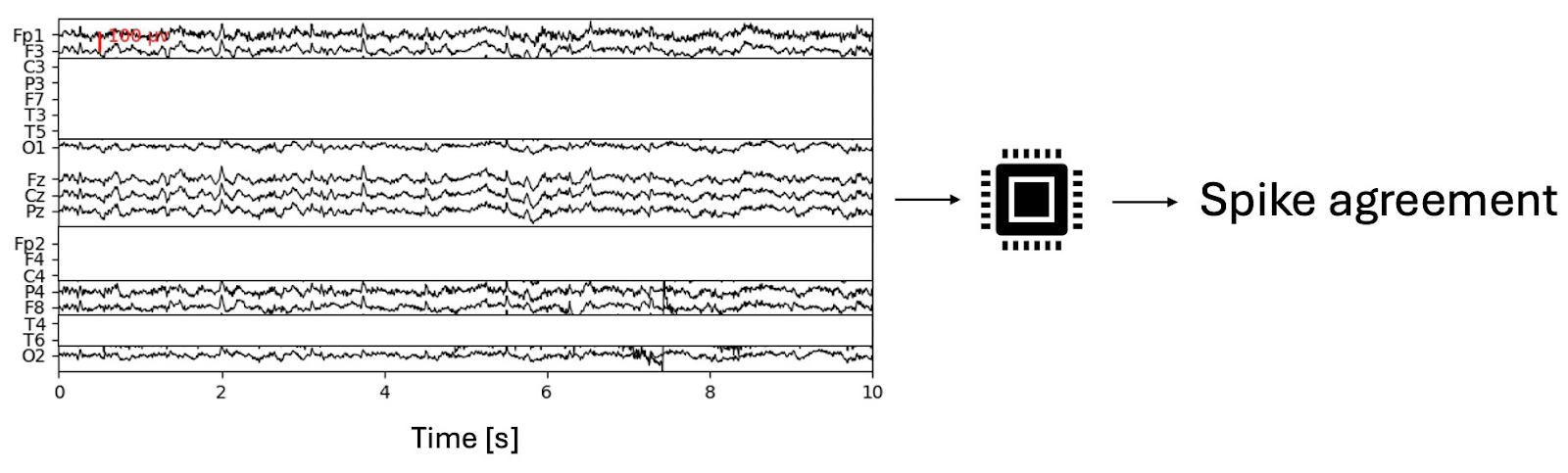

Supplement: Supplementary file 2 — Data S2. [file EPI-66-e114-s002.zip › EPI_18431_f3_Alkofer-FigS3.tiff]

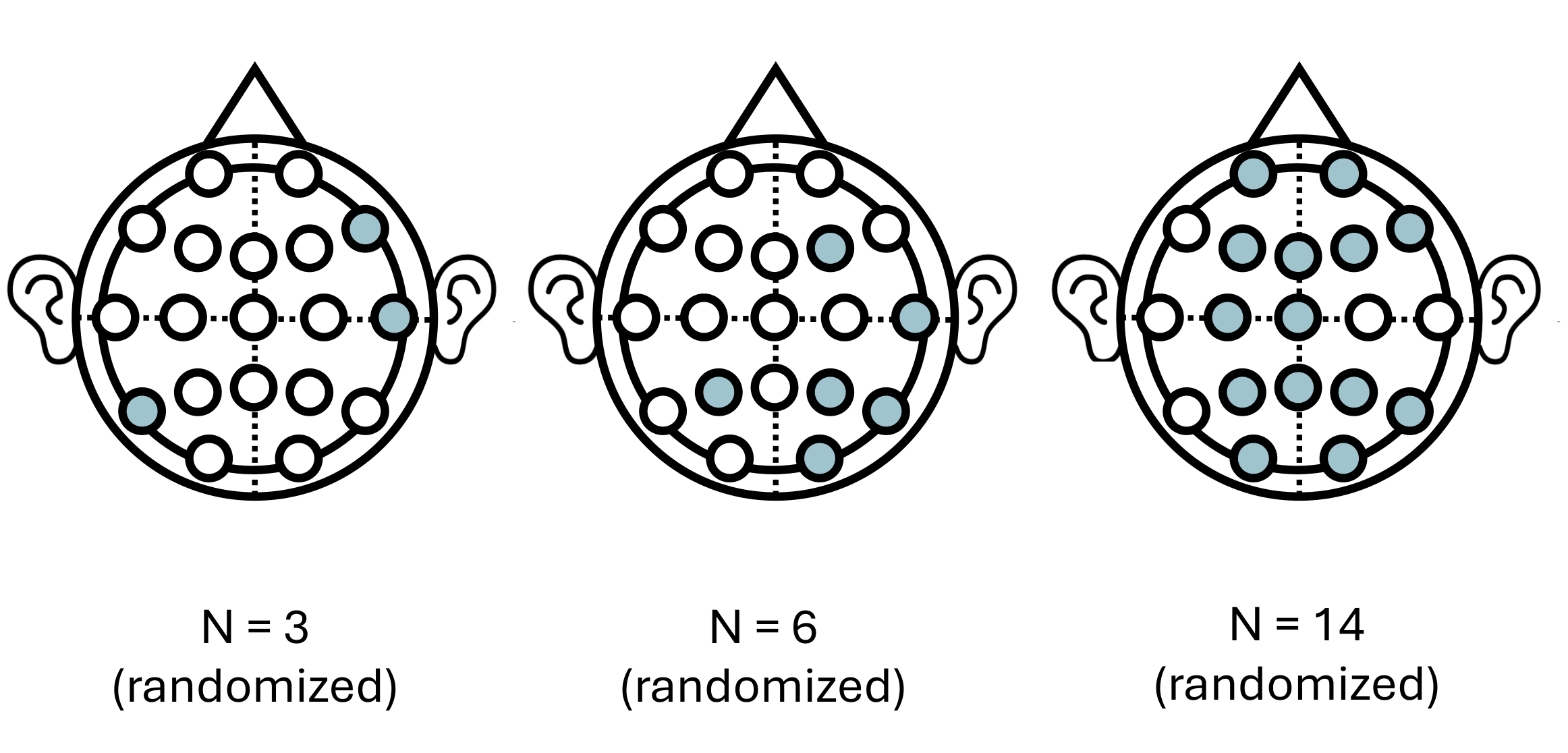

Supplement: Supplementary file 2 — Data S2. [file EPI-66-e114-s002.zip › EPI_18431_f4_Alkofer-FigS4a.tiff]

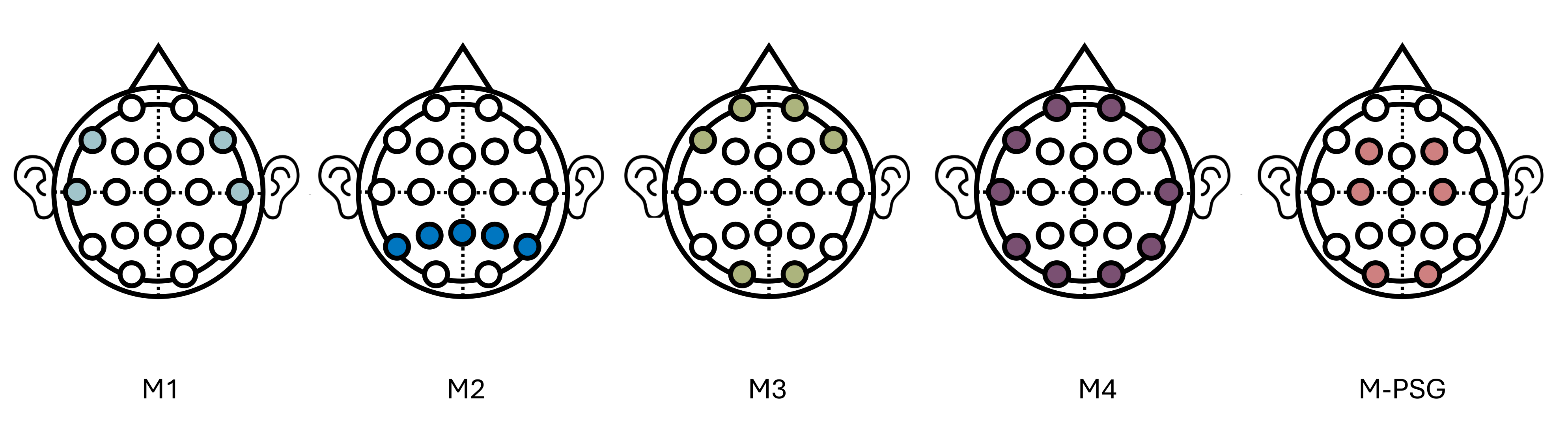

Supplement: Supplementary file 2 — Data S2. [file EPI-66-e114-s002.zip › EPI_18431_f4_Alkofer-FigS4b.tiff]

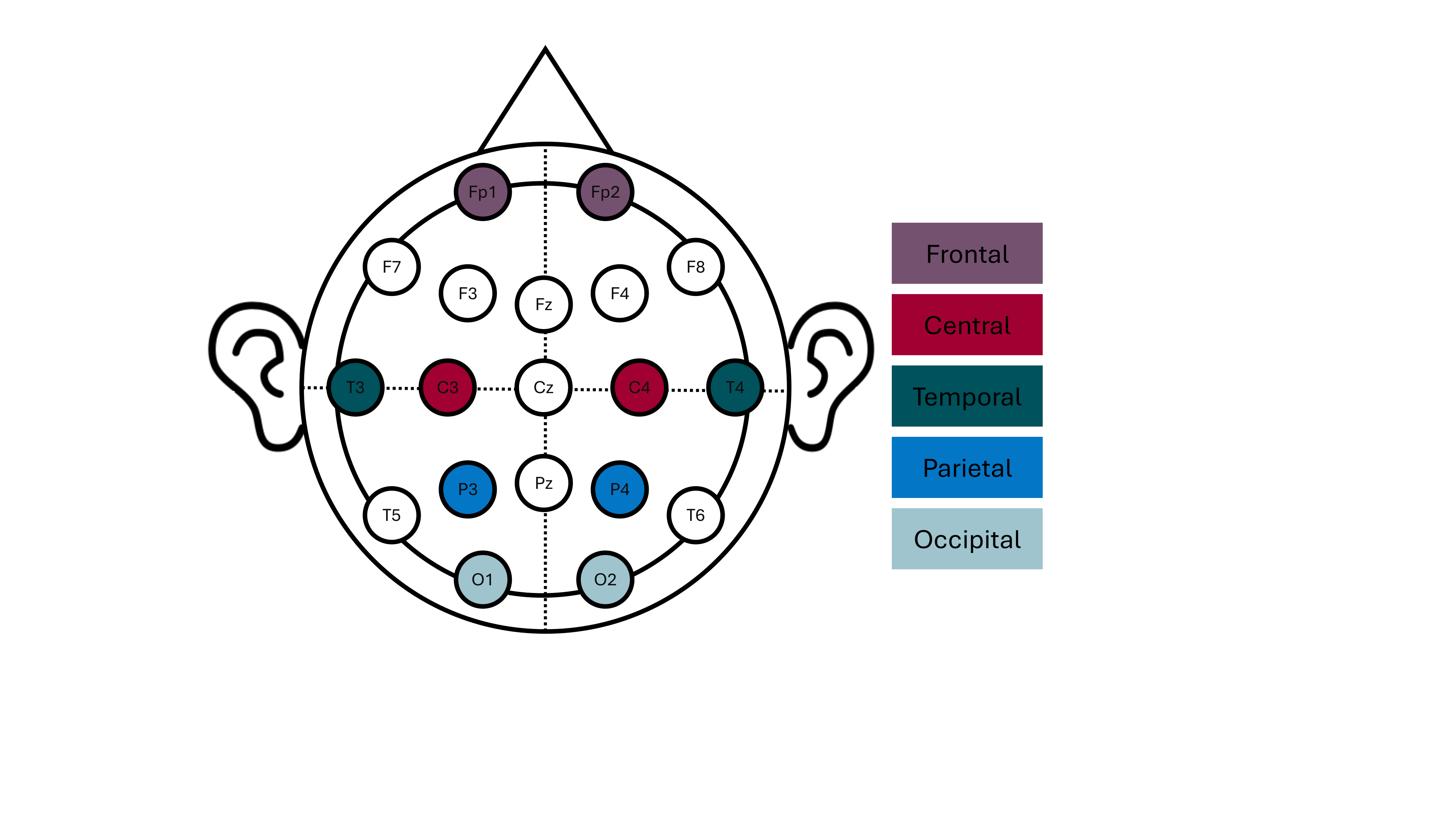

Supplement: Supplementary file 2 — Data S2. [file EPI-66-e114-s002.zip › EPI_18431_f4_Alkofer-FigS4c.tiff]

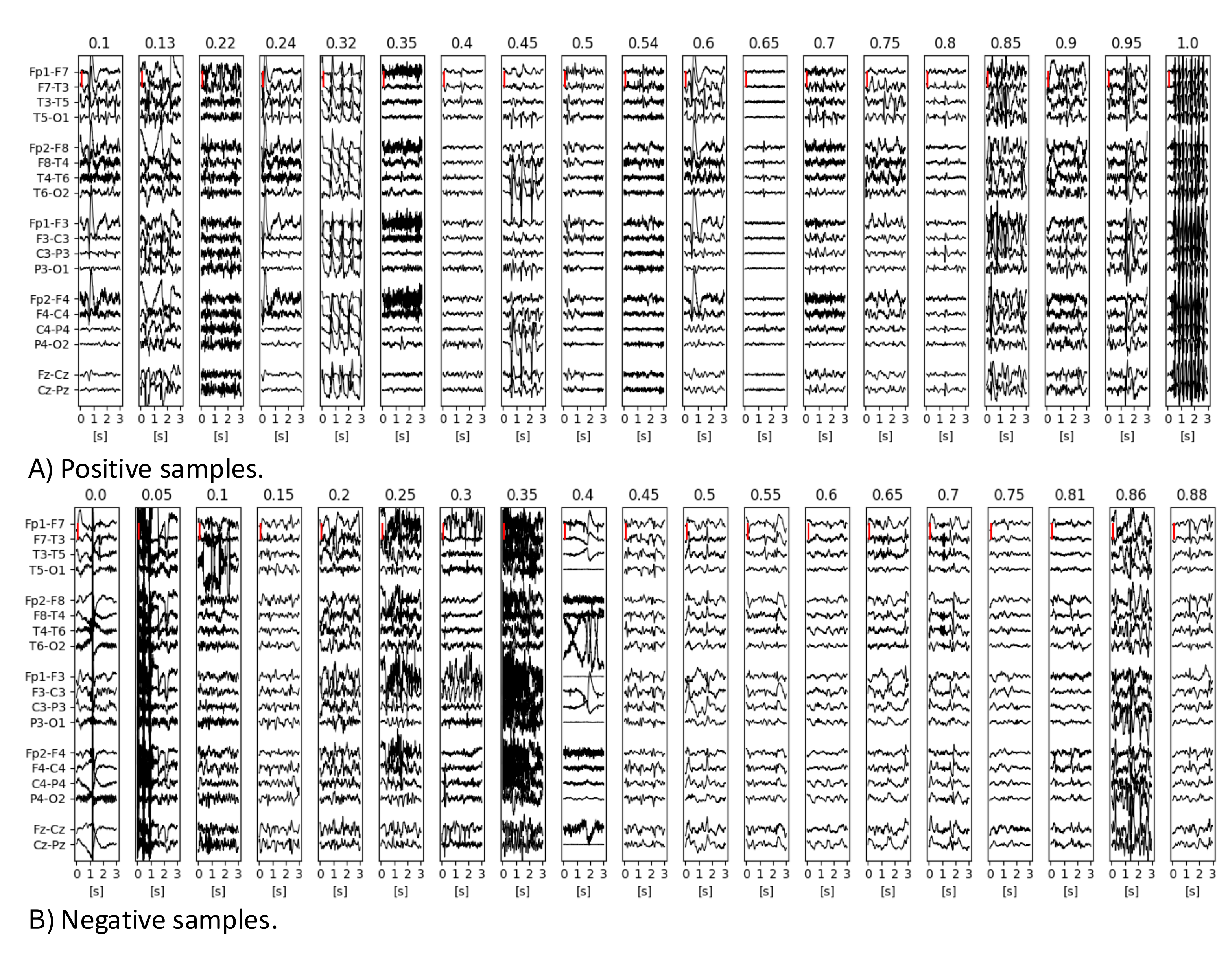

Supplement: Supplementary file 2 — Data S2. [file EPI-66-e114-s002.zip › EPI_18431_f6_Alkofer-FigS6.tiff]

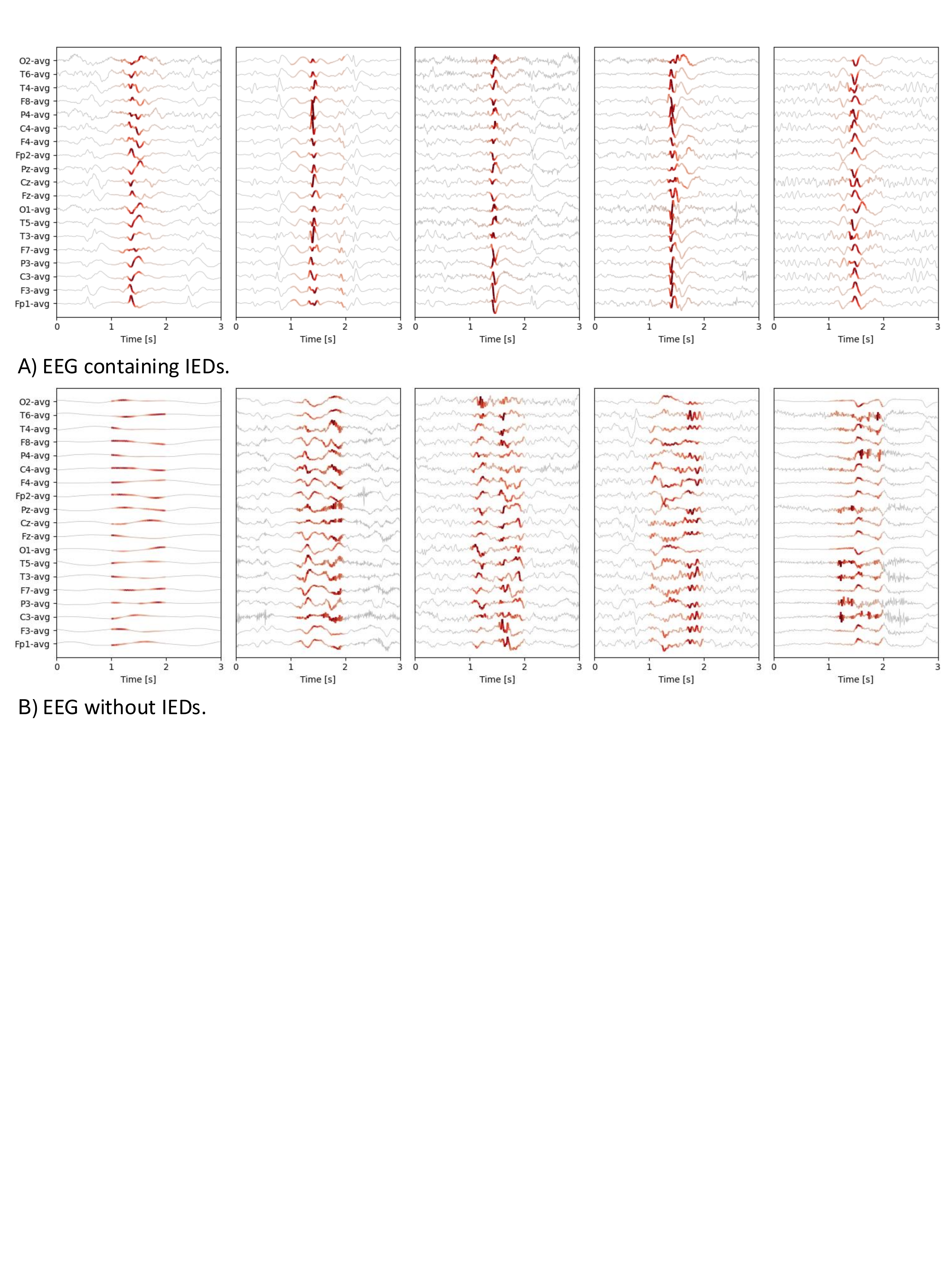

Supplement: Supplementary file 2 — Data S2. [file EPI-66-e114-s002.zip › EPI_18431_f7_Alkofer-FigS7.tiff]

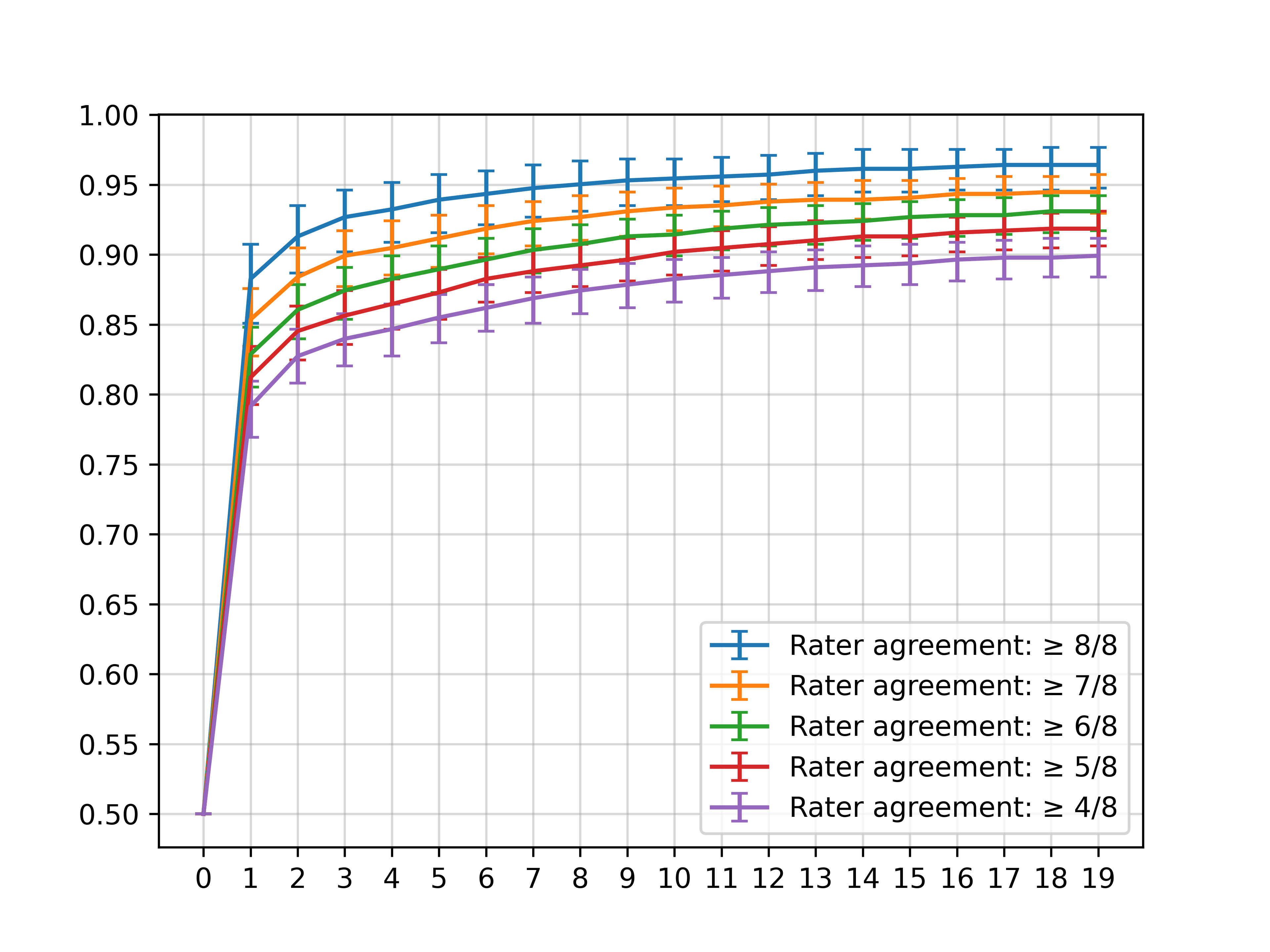

Supplement: Supplementary file 2 — Data S2. [file EPI-66-e114-s002.zip › EPI_18431_f8_Alkofer-FigS8.tiff]
